# Exome sequencing of a patient with suspected mitochondrial disease reveals a likely multigenic etiology

**DOI:** 10.1186/1471-2350-14-83

**Published:** 2013-08-16

**Authors:** William J Craigen, Brett H Graham, Lee-Jun Wong, Fernando Scaglia, Richard Alan Lewis, Penelope E Bonnen

**Affiliations:** 1 Department of Molecular and Human Genetics, Baylor College of Medicine, Houston, Texas, USA; 2 Department of Pediatrics, Baylor College of Medicine, Houston, Texas, USA; 3 Department of Ophthalmology, Baylor College of Medicine, Houston, Texas, USA; 4 Texas Children’s Hospital, Clinical Care Center, Houston, Texas, USA; 5 Human Genome Sequencing Center, Baylor College of Medicine, Houston, Texas, USA

**Keywords:** Encephalomyopathy, Lowe syndrome, OCRL, SETX, Diagnostics, Genocopy

## Abstract

**Background:**

The clinical features of mitochondrial disease are complex and highly variable, leading to challenges in establishing a specific diagnosis. Despite being one of the most commonly occurring inherited genetic diseases with an incidence of 1/5000, ~90% of these complex patients remain without a DNA-based diagnosis. We report our efforts to identify the pathogenetic cause for a patient with typical features of mitochondrial disease including infantile cataracts, CPEO, ptosis, progressive distal muscle weakness, and ataxia who carried a diagnosis of mitochondrial disease for over a decade.

**Methods:**

Whole exome sequencing and bioinformatic analysis of these data were conducted on the proband.

**Results:**

Exome sequencing studies showed a homozygous splice site mutation in SETX, which is known to cause Spinocerebellar Ataxia, Autosomal Recessive 1 (SCAR1). Additionally a missense mutation was identified in a highly conserved position of the OCRL gene, which causes Lowe Syndrome and Dent Disease 2.

**Conclusions:**

This patient’s complex phenotype reflects a complex genetic etiology in which no single gene explained the complete clinical presentation. These genetic studies reveal that this patient does not have mitochondrial disease but rather a genocopy caused by more than one mutant locus. This study demonstrates the benefit of exome sequencing in providing molecular diagnosis to individuals with complex clinical presentations.

## Background

The clinical presentation of mitochondrial disease is highly complex and variable, resulting in diagnostic challenges. Numerous manifestations include subacute necrotizing encephalomyelopathy (Leigh disease), cardiomyopathy, skeletal myopathy, anemia, intestinal pseudo-obstruction, liver failure, sensorineural hearing loss, diabetes and other endocrinopathies, renal tubulopathy, and growth failure. Chronic progressive external ophthalmoplegia (CPEO) is among the most commonly observed features, reported in nearly two-thirds of patients with mitochondrial myopathy. Molecular diagnosis is often pursued but the majority of these complex patients remain without a DNA-based diagnosis.

Pediatric-onset mitochondrial diseases are a diverse group and estimated to occur with an incidence of 1/5,000 [1]. Most cases (85–90%) are likely due to nuclear-encoded mutations, and autosomal recessive transmission is observed most commonly [1,2]. Despite their common occurrence, little is known about the molecular genetic basis of mitochondrial disorders.

We applied whole exome sequencing to identify pathogenic mutations in a patient with suspected pediatric-onset mitochondrial disease. The patient’s clinical presentation included classic hallmarks of mitochondrial disease: congenital cataracts, CPEO, early onset symmetric bilateral ptosis, progressive constitutional weakness, and ataxia. Exome sequencing revealed mutations in multiple genes that, when considered in aggregate, explain the patient’s phenotype.

## Methods

### Sample DNA

Informed consent was obtained from the subject and his parents for research and publication of clinical details according to a protocol approved by the Institutional Review Board for Baylor College of Medicine and Affiliated Hospitals. Genomic DNA was extracted from peripheral leukocytes and saliva according to standard protocols.

### Sequencing

We performed sequencing with a custom capture design called ‘VCROME’ developed at the BCM Human Genome Sequencing Center that enriches for approximately 20,000 genes comprising a total of 45 Mb of captured genomic sequence [3]. Coding exons in the Vega, Consensus Coding Sequence (CCDS) exons, and RefSeq annotation sets were targeted in addition to miRNAs from miRBase (release 10). In addition, 8 MB of untranslated regions (UTR) were enriched in this capture design. Probe pools were manufactured by Roche NimbleGen (Madison, WI). Samples were sequenced in a paired-end strategy on an Illumina HiSeq 2000 instrument with one lane per sample.

Targeted re-sequencing of both *SETX* and *OCRL* was completed for the propositus and each parent with primers SETXF1 TGACATTTCCTGTCAATGCCTC, SETXR1 GAATAACTCCCGGTGAGCCC, OCRLF1 GGACAGGAGGTAGCCAGAGAT, and OCRLR1 CGGCTTACTTTCTTATACTTGGC. The region was PCR-amplified and sequenced by di-deoxy terminator sequencing on an ABI 3730XL. All chromatograms were inspected manually, and genotype calls were based on bi-directional sequence reads with phred quality score > 20.

### Sequence data analysis and bioinformatics

Sequence data were aligned with BWA software to human reference sequence HG19 [4]. Duplicate reads were removed with the Picard program. Recalibration and realignment of the data were accomplished with GATK [5,6]. Single nucleotide variants (SNVs) were called with Samtools [7]. Small insertions and deletions (InDels) were determined by GATK. Quality control filtering of variants was based on coverage, strand bias, mapping quality, and base quality. Annotation of variants was conducted with internally developed Perl scripts. Prediction for potential functional consequences of variants was conducted with SIFT [8], PolyPhen2 [9], and PhyloP [10] using dbNSFP [11] and its scoring system which scales each of these metrics from 0 to 1 with 1 being most damaging. The evolutionary conservation of missense mutations was also determined by Genomic Evolutionary Rate Profiling (GERP) which approximates evolutionary constraint at a locus by maximum likelihood estimation [12,13]. The GERP++ score reported here is based on the alignment of 35 mammalian species and the maximum GERP score for this analysis is 6.18.

### Clinical description

The patient is a 33-year-old male, born to a then 20-year old mother following an uncomplicated pregnancy. His parents are first cousins of Persian descent. Infantile cataracts were noted at 3 months of age and the lenses removed shortly thereafter. Hypospadias (without cryptorchidism) was surgically repaired in infancy. His motor development was thought to be normal during early childhood, but CPEO, ptosis, and progressive distal muscle weakness developed in early adolescence, and ataxia and tremors became apparent as a young adult. A muscle biopsy was performed when the patient was an adolescent, but the results of the evaluation are not available; however, the family was told the results were inconclusive. Surgical correction of bilateral ptosis was not successful. Because of progressive ataxia, a magnetic resonance imaging (MRI) of the brain was performed when the patient was 25 years old and it demonstrated cerebellar atrophy.

The family history was notable for a younger sibling who died at 8 months of age of congenital heart disease. His mother has cerulean cataracts that do not interfere with her vision and were identified when she was in her 20’s. The maternal grandmother had cataract surgery when she was in her 50s. The patient’s mother had 3 siblings including a brother who underwent cataract surgery at 4 years of age and who died at 39 years of age from an accident. A second brother has no history of cataracts, while a sister also has cataracts. One of the sister’s two children has cataracts that have not required surgery. In the father’s family there is no history of cataracts.

The physical examination of the patient was notable for bilateral ptosis, complete CPEO with a small angle esotropia and a normal retinal exam, neck weakness, extremity weakness that was more prominent in the lower extremities, upper extremity tremors, and symmetrically markedly reduced reflexes.

Relevant clinical biochemical and molecular diagnostic testing performed at 30 years of age included plasma thymidine level, urine organic acids analysis, carbohydrate-deficient transferrin analysis, a complete metabolic panel, and TSH, all of which were normal. The blood lactate was increased at 2.6 mM (normal range 0.2-2.0 mM), as was creatine kinase at 279 U/L (normal range 55–170 U/L).

Genetic diagnostic testing included whole mitochondrial genome sequencing which detected one homoplasmic rare variant (m.3110C > T) in the 16S rRNA gene. This variant has been reported in Mitomap as a polymorphism ( http://www.mitomap.org/MITOMAP/). Additional diagnostic sequencing of three nuclear genes known to cause CPEO; *POLG*, *C10ORF2*, and *ANT1*, also found no deleterious mutations, and *OPA1* sequencing was also normal.

## Results

Exome sequencing of the proband resulted in 127X average coverage of targeted regions and 98% of targeted bases having at least 10 reads. Variant analysis revealed 17,328 total coding single nucleotide variants (SNVs) with a transition/transversion ratio of 3.3, and 188 total small insertions and deletions (InDels) (Table 1). Due to the patient’s clinical diagnosis of suspected pediatric-onset mitochondrial disease, exome sequence results were analyzed with an assumed recessive genetic model. Fifteen (15) homozygous or hemizygous SNVs and 4 homozygous InDels were not reported in dbSNP137. One of the SNVs is predicted to affect splicing. Four of the SNVs were non-synonymous missense mutations; of these two were predicted to damage protein function due to high conservation across species (Table 2).

**Table 1:** Summary of coding variants identified through exome sequencing

**A**									
**ALL**				**KNOWN**			**NOVEL**		
**Ti/Tv**	**Total**	**Het**	**Hom**	**Total**	**Het**	**Hom**	**Total**	**Het**	**Hom**
3.0	17328	10277	7051	17048	10012	7036	280	265	15
**B**									
**ALL**	**KNOWN**	**NOVEL**	**INSERTION**	**DELETION**	**HET**	**HOM**		**NOVEL HOM**	
427	342	85	228	199	239	188		4	

**A**: Single nucleotide variants and **B**: small insertions and deletions. Variants are listed as novel if not present in dbSNP137.

**Table 2:** Summary of novel non-synonymous coding homozygous variants in patient

**CHROM**	**POS**	**REF**	**ALT**	**Gene**	**Position**	**Variant type**	**Amino acid change**	**GERP**	**PhyloP**	**SIFT**	**Polyphen2**	**OMIM disorders**
9	135176191	C	T	SETX	Splice	Splice	-	4.88	-	-	-	Ataxia-ocular apraxia-2, 606002 (3); Amyotrophic lateral sclerosis4, juvenile, 602433 (3)
X	128695181	G	A	OCRL	Exon_CDS	Missense	E284K	5.44	1.00	1.00	0.99	Lowe syndrome, 309000 (3); Dent disease 2, 300555 (3)
4	86863251	C	T	ARHGAP24	Exon_CDS	Missense	R47C	5.11	1.00	0.90	0.94	-
X	3021820	G	A	ARSF	Exon_CDS	Missense	G374S	3.43	0.99	0.86	0.18	-
X	153524232	G	A	TKTL1	Exon_CDS	Missense	R7K	0.12	0.22	0.80	0.19	-
4	88536013	TAGCAGTGACAGCAGCAAC	T	DSPP	Exon_CDS	Deletion	-	0.409		-	-	Dentinogenesis imperfecta, Shields type II/III, 125490/125500 (3); Deafness,autosomal dominant 36, with dentinogenesis, 605594 (3);
10	33136818	TAA	T	C10orf68	Exon_CDS	Deletion	-	−0.964	-	-	-	-
15	42302337	CA	C	PLA2G4E	Exon_CDS	Deletion	-	1.58	-	-	-	-
14	98444454	TC	T	C14orf64	5’UTR	Deletion	-	3.87	-	-	-	-

Analysis of results of exome sequencing showed that the patient is homozygous for a splice site mutation in *senataxin* (*SETX*). This mutation, *SETX* c.5375-1G > A (GenBank Accession Number: NM_015046.5), is in the −1 position of exon 10 of the *SETX* transcript. *SETX* has a total of 24 exons encoding a protein of 2677 amino acids long and contains at its C terminus a seven-motif domain that places it in the superfamily 1 of helicases. Recessive mutations in *SETX* cause Spinocerebellar Ataxia, Autosomal Recessive 1 (SCAR1, OMIM 606002). Patients typically develop juvenile-onset progressive ataxia, and some but not all patients exhibit oculomotor apraxia, hence the condition has also been termed ataxia with oculomotor apraxia type 2 (AOA2). Other features include increases in serum alpha-fetoprotein and creatine kinase. The clinical phenotype previously reported for SCAR1 parallels the ataxia, tremor, cerebellar atrophy, and hyporeflexia observed in the patient described here, thus, the homozygous *SETX* splice site mutation likely accounts for the patient’s ataxia. Heterozygous missense substitutions in *SETX* can give rise to dominant juvenile amyotrophic lateral sclerosis (ALS4, OMIM 602433).

The patient also has several clinical features that have not been reported to occur in SCAR1. In particular, the patient does not have ocular apraxia but rather CPEO, ptosis, and a history of bilateral congenital cataracts, features not reported in a recent survey of 90 mutation positive cases of SCAR1/AOA2 [14]. Given this clinical picture, the possibility that more than one gene locus gave rise to the patient’s phenotype was entertained, and the identified variants were re-analyzed with both recessive and dominant genetic models. Further review of the list of genes harboring variants not found in public databases and predicted to be damaging to protein function failed to identify any genes that have been annotated as part of the mitochondrial proteome, but did reveal an additional missense variant in the known disease gene, *OCRL*. Mutations in *OCRL* cause Lowe Syndrome as well as Dent2 disease. The oculocerebrorenal syndrome of Lowe includes variable involvement of the eyes, brain, and kidneys that could possibly explain the patient’s historical congenital cataracts. The missense variant, *OCRL* c.850G > A; p.E284K (GenBank Accession Number: NM_000276.3), was identified in exon 10. Most persons with Lowe Syndrome have mutations in exons 10–23 and many of these are missense [15]. Two different tests for evolutionary conservation, GERP and PhyloP, both showed that this base position is highly constrained. The GERP test for evolutionary sequence conservation yielded a score of 5.44, which is the maximum or most constrained score possible (Table 2). PhyloP analysis also showed significant evidence for conservation (score = 1). Software programs that predict whether a missense mutation is damaging to protein function scored this variant with the most damaging score (Sift = 1, PolyPhen2 = 1). Additionally, this *OCRL* variant is not present in either dbSNP or HGMD.

There is substantial bioinformatics support that this mutation damages protein function and therefore would manifest as Lowe Syndrome in this male, however, we were unable to complete a full clinical assessment for Lowe Syndrome in the patient because the patient was not available for further clinical investigations. Likewise, due to a lack of tissue from the patient we were not able to confirm directly if the *SETX* c.5375-1G > A results in the loss of exon 10 or otherwise affects this protein.

**Figure 1:**
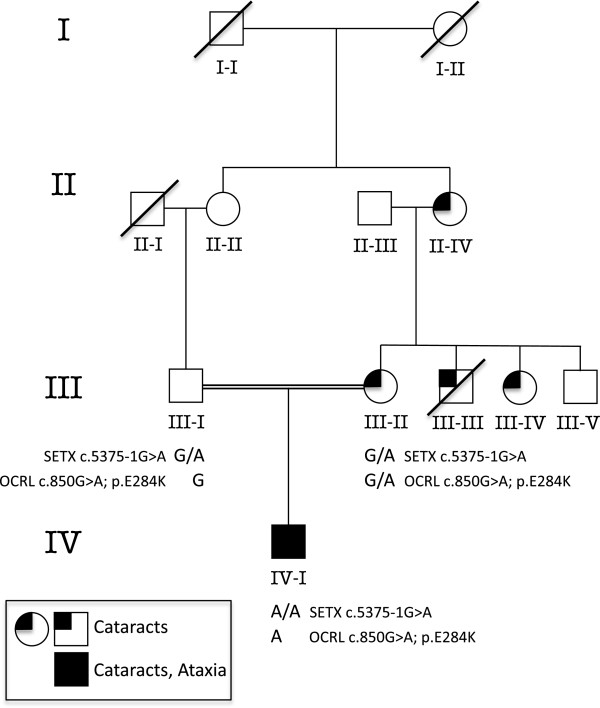
**Pedigree of patient with ataxia and congenital cataracts.** Four generations of the family of a proband with ataxia and congenital cataracts. Genotypes for the *SETX* mutation chr9:135,176,191, C > T and the *OCRL* mutation is chrX:128695181, G > A are shown for the proband and his parents. Several members of the maternal lineage were noted to have early onset cataracts.

DNA samples from each parent were sequenced. Each was heterozygous for the *SETX* splice site mutation, and the mother was found to be a carrier of the *OCRL* variant. The patient’s mother did not display the characteristic cortical flecks in the lens typical of Lowe Syndrome carriers, but instead she has bilateral cerulean cataracts that were first discovered when she was in her 20’s. The mother’s pedigree shows three other family members with cataracts, one who had an early age of onset necessitating surgery (Figure 1). The possibility of an independent locus segregating in this family that confers autosomal dominant cataract was evaluated. We queried the patient’s exome variants present in genes known to cause all inherited forms of cataract, reviewed in Huang *et al.* 2010 [16]. No variants that were predicted to damage protein function were identified in any known cataract gene. This includes four known cerulean cataract genes: *MAF*, *CRYG*, *CRYBB2*, and *GJA3.* Ascertainment of *CRYG* and *CRYBB2* coding region was 100% in the exome dataset. However, part of the first exon of *MAF* and the last exon of *GJA3* are both repeat rich and as a result was not targeted by exome capture. Attempts to sequence these gene regions by Sanger sequencing were unsuccessful.

## Discussion

We describe a patient with a complex clinical presentation including congenital cataracts, CPEO, ptosis, progressive systemic neuromuscular weakness, cerebellar atrophy, ataxia, and tremors, and the results of sequencing his exome. Exome sequencing revealed potentially pathogenic variants in two genes: *SETX* and *OCRL*, each of which may contribute to this complex phenotype. Recently, senataxin was found to harbor RNA/DNA helicase activity important in mRNA transcription, with an ability to unwind RNA/DNA hybrids formed behind the elongating RNA Polymerase II between the nascent RNA transcript and single-stranded DNA template. This activity is likely important in the termination of transcription at the polyA addition signal [17], and studies in yeast point to a similar 3’ processing function in noncoding RNAs [18], suggesting that perturbations in global gene expression may underlie the pleiotropic features of the disease.

This study represents the challenges in diagnosing patients with complex clinical phenotypes and the interpretation of exome sequence data, a technology that is moving rapidly into clinical diagnostics. This patient’s initial diagnosis of mitochondrial disease was suggested by his findings and offered without the benefit of genome-wide sequence data. Exome sequencing revealed that his complex phenotype reflects a complex genetic etiology in which no single gene explained the complete clinical presentation. The mutation found in *SETX* partially explains his clinical diagnosis, while the pathogenicity of the *OCRL* missense variant remains undetermined. There is strong bioinformatics support for pathogenicity, however, the patient was not available for further clinical investigations and the patient’s mother did not exhibit the classic ocular phenotype of heterozygotes of Lowe Syndrome mutations. We considered the possibility of an independent locus causing the congenital cataracts in this patient. Exome data for genes known to cause dominantly inherited cataracts were queried and no variants with predicted pathogenicity were found. In summary, exome sequencing improves the diagnostic reservoir, and some patients may require further clinical and/or functional studies to achieve a complete diagnosis.

## Conclusions

Exome sequencing studies identified mutations in multiple independent loci in a patient with typical features of mitochondrial disease including infantile cataracts, CPEO, ptosis, progressive distal muscle weakness, and ataxia who carried a diagnosis of mitochondrial disease for over a decade. Sequencing revealed a homozygous splice site mutation in *SETX*, which is known to cause Spinocerebellar Ataxia, Autosomal Recessive 1 (SCAR1), as well as a missense mutation in a highly conserved position of the *OCRL* gene, which causes Lowe Syndrome and Dent Disease 2. This patient’s complex phenotype reflects a complex genetic etiology in which no single gene explained the complete clinical presentation. These genetic studies reveal that this patient does not have mitochondrial disease but rather a genocopy caused by more than one mutant locus. This study demonstrates the benefit of exome sequencing in providing molecular diagnosis to individuals with complex clinical presentations.

## Competing interests

The authors declare no competing interests.

## Authors’ contributions

PEB participated in study design, drafting of the manuscript, collection and analyses of data. WC participated in collection of samples and data interpretation. WC, BG, RL, FS clinically evaluated the patient and family. LW conducted diagnostic screening. All authors read and approved the final manuscript.

## Pre-publication history

The pre-publication history for this paper can be accessed here:

http://www.biomedcentral.com/1471-2350/14/83/prepub
